# Antimicrobial Photodynamic Therapy Using Encapsulated Protoporphyrin IX for the Treatment of Bacterial Pathogens

**DOI:** 10.3390/ma17081717

**Published:** 2024-04-09

**Authors:** Natalia Izquierdo, Enrique Gamez, Teresa Alejo, Gracia Mendoza, Manuel Arruebo

**Affiliations:** 1 Aragon Health Research Institute (IIS Aragon), 50009 Zaragoza, Spain; nizquierdo@iisaragon.es (N.I.); engahe@unizar.es (E.G.); gmendoza@iisaragon.es (G.M.); 2 Instituto de Nanociencia y Materiales de Aragón (INMA), CSIC-Universidad de Zaragoza, 50009 Zaragoza, Spain; teresaalejo@gmail.com; 3 Department of Chemical Engineering, University of Zaragoza, Campus Río Ebro-Edificio I+D, C/Poeta Mariano Esquillor S/N, 50018 Zaragoza, Spain; 4 Department of Pharmacology and Physiology, Forensic and Legal Medicine, Veterinary Faculty, University of Zaragoza, 50009 Zaragoza, Spain

**Keywords:** protoporphyrin IX, photodynamic therapy, encapsulation, PLGA, *Staphylococcus aureus*, photobleaching

## Abstract

Herein, we report on the antimicrobial photodynamic effect of polymeric nanoparticles containing the endogenous photosensitizer protoporphyrin IX. Compared to equivalent doses of the free photosensitizer, we demonstrated that the photodynamic antimicrobial efficacy of PLGA (polylactic-co-glycolic acid) nanoparticles containing protoporphyrin IX (PpIX) against pathogenic *Staphylococcus aureus* (*S. aureus*) is preserved after encapsulation, while photobleaching is reduced. In addition, compared to equivalent doses of the free porphyrin, we show that a reduction in the cytotoxicity in mammalian cell cultures is observed when encapsulated. Therefore, the encapsulation of protoporphyrin IX reduces its photodegradation, while the released photosensitizer maintains its ability to generate reactive oxygen species upon light irradiation. The polymeric nanoencapsulation promotes aqueous solubility for the hydrophobic PpIX, improves its photostability and reduces the cytotoxicity, while providing an extended release of this endogenous photosensitizer.

## 1. Introduction

In antimicrobial photodynamic therapy (aPDT), a photosensitizer triggers local light-activated photochemical reactions, produced by electron or energy transfer. Those reactions produce the inactivation of pathogens based on the generation of superoxide anions and other reactive oxygen species (^•^OH, O_2_^−^, H_2_O_2_) (type I reactions based on electron transfer) or by the production of singlet molecular oxygen (^1^O_2_) (type II reactions based on energy transfer). The spatio-temporal control of the photochemical reactions involved is one of the main advantages of this therapy by reducing off-target effects, which has facilitated the clinical management of different oral and skin conditions associated with microbial colonization. Pathogenic viruses, fungi, protozoa, and bacteria, alone and as polymicrobial infections have been successfully eradicated using aPDT [1,2]. After the irradiation of an endogenous or exogenous photosensitizer and under the presence of oxygen, a cascade of antimicrobial mechanisms is activated due to the generation of reactive oxygen species (ROS) including the following: membrane depolarization and increased fluidity and permeability, membrane breakage, and membrane phospholipid rearrangement and peroxidation, which are associated with the ROS generated by type I reactions (i.e., ^•^OH, O_2_^−^, H_2_O_2_) [2]. The main product generated by type II reactions (i.e., ^1^O_2_) is responsible for additional cytotoxic effects including oxidative damage to proteins, lipids, and nucleic acids [3]. The chances for bacteria to develop resistance mechanisms against aPDT are reduced because multiple ROS are simultaneously generated and those species have short lifetimes, usually in the millisecond range or less [4], and short intracellular diffusion lengths from their point of generation (in the hundreds of nm range) [5]. Therefore, the probabilities for bacteria to produce phenotypic modifications against aPDT are low considering the short ROS timespan and the long distance from their point of generation to the intracellular machinery responsible for the genetic transcription and translation into antioxidative stress enzymes or to other counteracting mechanisms. Despite this, it has been reported that bacteria can upregulate the expression of their antioxidant enzymes against type I produced species; to the best of our knowledge, an enzyme-mediated defensive system against type II generated species has not been reported yet [6]. In addition to the spatio-temporal therapeutic control mentioned above and to the reduced opportunities for bacteria to develop resistance, aPDT constitutes a noninvasive approach having a broad-spectrum antimicrobial activity. It also shows reduced long-term side effects, triggers non-specific antimicrobial mechanisms, and demonstrates synergetic effects with other antimicrobial technologies (i.e., with photothermal therapy, antibiotics, chemotherapy, metal nanoparticles, immunotherapy, etc.) [7,8]. However, aPDT shows some drawbacks including the limited penetration depth in the tissues reached by the VIS or even the NIR light used, the non-selective oxidative damage to the entire irradiated area, potential skin photosensitivity or photosensitizer photobleaching, among others [9]. To solve some of those limitations, the encapsulation of photosensitizers within organic and inorganic nanoparticles (NPs) has been extensively used [10]. Their encapsulation within nanoparticles allows an improved aqueous solubility and targeted or sustained delivery towards pathogenic bacteria. Nanoencapsulation has also been used to prevent photobleaching, to trigger the delivery of photosensitizers using endogenous or exogenous stimuli, to enhance their permeation through the tissues, to augment bacterial cell binding, to increase ROS production, and so on [10,11,12,13]. 

Protoporphyrin IX (3,7,12,17-Tetramethyl-8,13-divinyl-2,18-porphinedipropionic acid) is one of the clinically approved photosensitizers used in the treatment of different skin conditions (e.g., actinic keratosis, acne, rosacea, etc.), some cancers (bronchial, esophageal, Bowen’s disease, and other premalignant lesions) and in diagnosis (by the photodynamic detection of its fluorescence when accumulated in cancer cells during fluorescence-guided surgery) [14,15]. Protoporphyrin IX is ubiquitous in all human cells, acting as an intermediate product of heme (i.e., iron protoporphyrin IX); however, due to its high hydrophobicity, it can cause hepato- and biliary toxicity when externally administered [14]. To reduce its toxicity, aminolevulinic acid (ALA) is used as a topical exogenous precursor, which is intracellularly converted into protoporphyrin IX. Also, hydrophilic formulations have been developed to increase its bioavailability (e.g., methyl aminolevulinate). The encapsulation of protoporphyrin IX within nanocarriers has also been used to reduce its toxicity [16], to enhance its bioavailability [17], to improve skin penetration [18], to enhance its ROS-producing ability in physiological media [19], and to improve its efficacy compared to equivalent doses of the free photosensitizer [20,21].

In aPDT, the encapsulation of protoporphyrin IX within polymeric carriers based on poly(ethylene glycol) and poly(β-amino ester) has demonstrated improved biofilm permeation and accumulation, and the ability to eradicate antibiotic-resistant subcutaneous *staphylococcal* infections in vivo [22]. Moreover, cholesterol-modified poly(ethylene glycol)-based micelles containing protoporphyrin IX have also shown enhanced hydrophobic interactions with the outer membrane of Gram-negative bacteria and extended antimicrobial action against both Gram-positive and Gram-negative bacteria [23]. In other recent examples, a polymeric carrier (chitosan) has been used to avoid the cytotoxicity and hemolytic activity of polycations (e.g., polyethylenimine) bound to protoporphyrin IX, used to electrostatically interact with bacteria [24]. Polymeric nanocarriers based on Pluronic^®^ F-127 containing protoporphyrin IX and gallium have also demonstrated not only antibiofilm activity but also the ability to eradicate intracellular pathogens in in vitro models of infection [25]. 

Polylactic-co-glycolic acid (PLGA)-based nanoparticles are widely used as delivery vectors of many therapeutics, including photosensitizers, for their ability to biodegrade its ester bonds by hydrolysis, rendering endogenous lactic and glycolic acids, which are rapidly metabolized by the Krebs cycle. Their sustained-release ability, protection capability, augmented solubility, stability for loaded hydrophobic drugs, and degradation tunability depending on the lactic acid and glycolic acid contents and molecular weights, are properties extensively used in many biomedical applications. PLGA nanoparticles have been used in the encapsulation of protoporphyrin IX (or ALA) to promote its in vivo transdermal delivery [26] and to improve the phototoxicity against cancer cells including murine melanoma cell cultures [27], human prostate cancer cells [28], human skin squamous cell carcinoma cell lines [29], mouse mammary tumor cell lines [30], etc. However, to the best of our knowledge, the antimicrobial action of free protoporphyrin IX and protoporphyrin-IX-loaded PLGA nanoparticles (as solid matrix systems) has not been reported to date. Herein, we have evaluated their photodynamic antimicrobial efficacy against pathogenic bacteria (i.e., *S. aureus*) commonly associated with skin and soft tissue infections, and the reduction in cytotoxicity when encapsulated vs. the free compound at the same doses in mammalian cell cultures. Therefore, the nanoencapsulation of protoporphyrin IX within polymeric matrices increases its bioavailability and consequently its therapeutic efficacy.

## 2. Materials and Methods

### 2.1. Materials

PLGA-Amine terminated/PLGA-NH_2_ (50:50) was purchased from GenoChem World SL (Valencia, Spain). Dichloromethane (DCM, >99.8%), dimethyl sulfoxide (DMSO, >99%), Mowiol^®^ 4–88, Tween^®^ 20, phosphate-buffered saline (PBS), dihydrorhodamine 123 (DHR123), protoporphyrin IX (PpIX), indocyanine green (ICG), and potassium chloride (KCl, 99.0–100.5%) were purchased from Sigma-Aldrich (Darmstadt, Germany). Tryptone soy agar (TSA) was purchased from Laboratorios Conda-Pronadisa SA (Madrid, Spain). Tryptone soy broth (TSB) was purchased from VWR Chemicals. *S. aureus* ATCC 29213 was obtained from Ielab (Alicante, Spain). Human dermal fibroblasts (NHDF-Neo, Lonza, Basel, Switzerland) were used in order to evaluate the potential cytotoxicity of PpIX and the synthetized particles. High-glucose Dulbecco’s modified Eagle’s medium (DMEM; DMEM w/stable glutamine) and antibiotics−antimycotics (60 μg/mL penicillin, 100 μg/mL streptomycin, and 0.25 μg/mL amphotericin B) were supplied by Biowest (Nuaille, France). The medium was supplemented with 10% (*v*/*v*) fetal bovine serum (FBS) from Gibco (Thermo Fisher Scientific, Waltham, MA, USA). The Blue^®^ Cell Viability Assay, used to evaluate the dose-dependent cytotoxicity, was purchased from Abnova (Taipei, Taiwan). 

### 2.2. Photosensitizer Selection

The DHR123 fluorescent probe was employed to quantify ROS production after photodynamic activation of the FDA-approved photosensitizers ICG and PpIX. For the ICG ROS production analysis, DHR123 (1.6 μM) and ICG (40 ppm) in ethanol were prepared. Three replicates were measured using an LS 55 fluorescence spectrophotometer (Perkin Elmer, Waltham, MA, USA) before and after 3 min of irradiation with an 808 nm diode laser (6 × 8 mm^2^ spot size; Optilas model MDL-III-808-2W, Changchun New Industries Optoelectronics Technology Co., Ltd., Changchun, China) using a power controller (Model PD300-3W, Ophir Laser Measurement Group, Logan, UT, USA) at 1 or 0.5 W/cm^2^. For the evaluation of PpIX ROS production, DMSO was used to solubilize PpIX mixed with MilliQ water before irradiating with a 532 nm diode laser at 0.5 W/cm^2^ (6 × 8 mm^2^ spot size; Optilas model MGL-II-532-500 mW, Changchun New Industries Optoelectronics Technology Co., Ltd., Changchun, China). Control measurements were carried under the same experimental conditions but without laser irradiation in order to verify that no ROS were generated in the absence of irradiating light. In order to decouple photothermal from photodynamic effects, control measurements were also performed to evaluate whether any temperature increase would contribute to a cellular viability decrease or ROS generation using the same photosensitizer concentrations. Photosensitizer photobleaching was studied by collecting the UV–Vis spectra before and after exposing the corresponding photosensitizers to their respective laser wavelengths (808 nm for ICG, 532 nm for PpIX) for 5 min at a 0.5 W/cm^2^ irradiance.

### 2.3. Protoporphyrin-IX-Loaded PLGA Nanoparticles Synthesis and Characterization

The single-emulsion-solvent evaporation technique was used to prepare PpIX-loaded PLGA nanoparticles. Briefly, 2.5 mL of PLGA solution (0.4% *w*/*v*) was prepared by dissolving the polymer in DCM mixed with 200 µL of PpIX solution in DMSO (0.08% *w*/*v*) to form the organic phase. Then, 10 mL of MOWIOL^®^ 4–88 solution in Milli-Q water (5% *w*/*v*) was added and then sonicated in an ice bath, using an ultrasonic probe of 3.2 mm in diameter (Digital Sonifier 450, Branson, MI, USA) during three cycles of 25 s each, with an amplitude of 40% in order to create the o/w emulsion. The samples were maintained under stirring for 3 h at 600 rpm to allow solvent evaporation and then centrifuged (Heraeus Megafuge 16R, Thermo Fisher Scientific) at 10,000 rpm for 15 min. The supernatant was carefully decanted, and subsequently, 10 mL of Milli-Q water was added to each sample. For the nanoparticle resuspension, the tubes were sonicated (Digital Ultrasonic Cleaner) for 1 min. The suspension underwent two additional rounds of centrifugation, to remove non-encapsulated PpIX. After completing the third centrifugation, the pellet was resuspended in 2 mL of Milli-Q water, and the samples were stored in light-protected tubes at 4 °C. 

Nanoparticle tracking analysis (NTA) was carried out in a Nanosight NS300 (Malvern, UK) to evaluate the hydrodynamic diameter and NP concentration. The zeta potential of the nanoparticles, indicative of their surface charge density, was measured with a Brookhaven 90 Plus instrument by adding 1.5 mL of 1 mM KCl at pH 7.4 to 100 μL of the resulting nanoparticle suspensions. Each sample underwent three measurements.

Nanoparticle concentration was calculated by mass balance. Briefly, three measured volumes of the resulting nanoparticle suspension were weighted before and after solvent evaporation (at 37 °C until no weight changes were observed), and concentration was calculated as averaged ratios between the dried mass (in mg) and the volumes evaporated (in mL).

The morphological analysis of the nanoparticles was conducted using transmission electron microscopy (TEM) with a FEI Tecnai T20 microscope operating at an acceleration voltage of 200 kV using a cryogenic workstation. Before observation, a droplet of the aforementioned suspension was dispensed on a 200-mesh copper grid and desiccation was allowed under ambient conditions. Nanoparticle dimensions were evaluated through the utilization of DigitalMicrograph software v3.5 and diameters were measured (N = 100) using the ImageJ software v1.54 (ImageJ, US. NIH, Bethesda, MD, USA).

The PpIX loading in the nanoparticles was quantified by UV–Vis spectrophotometry (Jasco V670, Jasco Applied Science, Eschborn, Germany) at a wavelength of 407 nm by dissolving 50 µL of the PpIX-loaded NPs in 600 µL of DMSO. To eliminate any potential interference, a baseline of the same amount of the dissolved non-loaded polymer was subtracted.

The drug loading (DL) and the encapsulation efficiency (EE) were calculated using Equations (1) and (2), respectively:

(1) $$DL\left(\%\right)=\frac{\mathrm{mass}\;\mathrm{of}\;\mathrm{entrapped}\;\mathrm{photosensitizer}\;(\mathrm{mg})}{\mathrm{total}\;\mathrm{mass}\;\mathrm{of}\;\mathrm{photosensitizer}\;\mathrm{loaded}\;\mathrm{nanoparticles}\;(\mathrm{mg})}\times 100$$

(2) $$EE\left(\%\right)=\frac{\mathrm{mass}\;\mathrm{of}\;\mathrm{entrapped}\;\mathrm{photosensitizer}\;(\mathrm{mg})}{\mathrm{mass}\;\mathrm{of}\;\mathrm{photosensitizer}\;\mathrm{added}(\mathrm{mg})}\times 100$$

To assess drug release kinetics, samples were prepared by combining 40 µL of PpIX-loaded NPs with 960 µL of PBS containing 2 wt.% Tween^®^ 80 to allow drug release due to the high hydrophobicity of the PpIX. At specified time points, three samples were collected and subjected to centrifugation at 12,500 rpm for 15 min. The supernatant was collected and analyzed using UV–Vis spectrophotometry (Jasco V670, Jasco Applied Science, Eschborn, Germany) after subtracting the baseline obtained for the non-loaded polymeric NPs.

### 2.4. In Vitro Biological Analyses

The antibacterial activity of free PpIX was evaluated against *S. aureus* ATCC 29213, used as a model of Gram-positive bacteria. The microorganism was cultured overnight in TSB at 37 °C under continuous shaking (150 rpm) until the stationary growth phase was reached (10^9^ colony-forming units per mL (CFU/mL)). The inoculum was then added to tubes containing varying amounts of PpIX (0.5–10 ppm) dissolved in TSB with 2% DMSO. Positive controls included untreated *S. aureus* and an assay with just 2% DMSO (without PpIX) to confirm the lack of antimicrobial activity in the latter organic solution. After 1 h of incubation at 37 °C and 150 rpm shaking, the samples were irradiated using a laser with an irradiance of 0.5 W/cm^2^ for 5 min, using a 532 nm wavelength laser diode (6 × 8 mm^2^ spot size; Optilas model MGL-II-532-500 mW, Changchun New Industries Optoelectronics Technology Co., Ltd., Changchun, China). In parallel, identical samples were kept in the dark to assess the potential toxicity of the photosensitizers at the doses used and in the absence of light. Following irradiation, the standard serial dilution method was employed to determine viable bacteria for both irradiated and non-irradiated samples. Samples and controls were cultured on TSA plates and incubated overnight at 37 °C. Then, CFUs were quantified with an automatic colony counter (aCOLyte 3, Synbiosis, Cambridge, UK). All experiments were conducted at least three times. 

In order to evaluate the antimicrobial action of PpIX-loaded NPs, equivalent doses of the free photosensitizer were used (i.e., in the range 0.5–10 ppm). Positive controls included samples of untreated bacteria and samples containing the same amount of empty nanoparticles. All experiments were conducted in triplicate.

For the cytotoxicity study, fibroblasts were cultured in Dulbecco’s Modified Eagle Medium (DMEM) containing L-glutamine (2 mM) and supplemented with FBS (10% *v*/*v*) and antibiotics–antimycotics (1% *v*/*v* penicillin–streptomycin–amphotericin B) at 37 °C in a 5% CO_2_ atmosphere. Cells were seeded in 96-well plates at a density of 6000 cells/well. After 24 h, the culture medium was replaced with fresh medium containing the corresponding concentrations of PpIX, PpIX-loaded NPs, or empty NPs. For the evaluation of PpIX, a stock solution (1500 ppm) in DMSO was prepared and diluted with DMEM to reach the desired tested concentrations (0.5, 1.0, and 2.0 ppm). For PpIX-loaded NPs and empty NPs, NP dispersions in water were diluted with DMEM to reach the final tested concentrations (0.5, 1.0, and 2.0 ppm of PpIX in PpIX-loaded NPs). In both cases, the maximum DMSO or water concentrations used (without PpIX or NPs) were separately tested to check that DMSO or solvent did not affect cellular viability (results not shown). After incubation for 1 and 24 h, the Blue^®^ Cell Viability Assay was performed to correlate viability with the cell metabolism. Fluorescence was read (λex/λem 530 nm/590 nm) in a Varioskan LUX microplate reader (Thermo Fisher Scientific, USA). Viability was calculated by data interpolation assigning 100% viability to control samples (cells without any treatment).

### 2.5. Statistical Analyses

All data are reported as mean ± SD. Two-way analysis of variance (ANOVA) was used to statistically analyze the experimental data (GraphPad Prism 8, San Diego, CA, USA). Statistically significant differences were considered when *p* ≤ 0.01. 

## 3. Results

### 3.1. Photosensitizer Selection 

**Figure 1:**
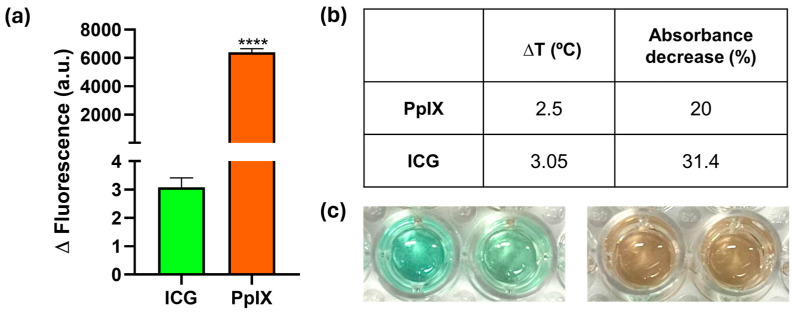
Selection and characterization of the photosensitizer: (**a**) Changes in fluorescence intensity observed following irradiation of the photosensitizer at an intensity of 0.5 W/cm^2^ for a duration of 3 min in the presence of the DHR probe. Experiments were performed in triplicate (n = 3; **** *p*-value < 0.0001); (**b**) Temperature increase and reduction in the absorbance at 407 and 793 nm (for PpIX and ICG, respectively) after irradiation at 0.5 W/cm^2^ for 3 min (∆T experiments) and 5 min (absorbance decrease during photobleaching experiments); (**c**) Optical images of accelerated photobleaching effects on a 40 ppm ICG solution (**left**) and a 40 ppm PpIX solution (**right**). The left well for each photosensitizer was not irradiated, and the right well was irradiated at 0.5 W/cm^2^ for five minutes.

The potential of the photosensitizers ICG and PpIX for aPDT was initially evaluated in terms of ROS production and photobleaching in order to select one of them for the subsequent experiments. Figure 1a shows the fluorescence emission using the DHR123 probe for both photosensitizers. This non-fluorescent probe is oxidized by different ROS (such as peroxide and peroxynitrite) and produces its fluorescent form (rhodamine 123) [31]. After irradiating the samples at the corresponding wavelengths using the same fluence (90 J/cm^2^), a significantly superior fluorescence was observed for PpIX (2000 times higher, Figure 1a). A superior fluorescence lifetime and quantum yield of PpIX [32] compared to ICG [33] might be responsible for the superior ROS production observed for the former. The temperature increase after the 3 min of irradiation used (0.5 W/cm^2^) was monitored as well as the reduction in the absorbance of the photosensitizers tested (Figure 1b). In order to evaluate whether the temperature increase after irradiating could damage bacteria in the subsequent in vitro antimicrobial photodynamic assays, the temperature increase was measured after 3 min of irradiation (the same conditions used in the bactericidal study). The temperature increase was lower than 3 °C, which did not produce any reduction in the bacterial cell counts. Therefore, a potential photothermal effect caused by the irradiating conditions was ruled out. To evaluate the photothermal stability of both photosensitizers, the decrease in their maximum absorbance (Soret band at 407 nm for PpIX and 793 nm for ICG) was tested after 5 min of irradiation. A superior decrease in the absorbance measured was observed for the ICG; thus, photobleaching was more pronounced for ICG under those conditions (Figure 1c). Taking this result into account, together with the ROS production measured (Figure 1a), PpIX was chosen over ICG for the following antimicrobial studies. Light-induced photobleaching has been previously analyzed for ICG and fitted to a first-order kinetic reaction [34]. Photobleaching is also reported for PpIX but following a second-order reaction [35] which could explain the reduced absorbance decrease observed for this photosensitizer at the concentrations tested. Likewise, Noghreiyan et al. [36] showed in an in vitro comparative photodynamic study, a superior action of PpIX over ICG at the same concentrations on the elimination of breast cancer cell lines (i.e., MCF-7).

### 3.2. PpIX-Loaded PLGA Nanoparticles Synthesis and Characterization

Once the best photosensitizer was chosen, PLGA nanoparticles loaded with PpIX were fabricated and characterized in order to protect PpIX from degradation and, thus, lengthen its lifespan and achieve a sustained release. Hence, the biological effects of PpIX would potentially last longer, avoiding the microbiological recolonization of infected tissues and side effects after treatment. 

**Figure 2:**
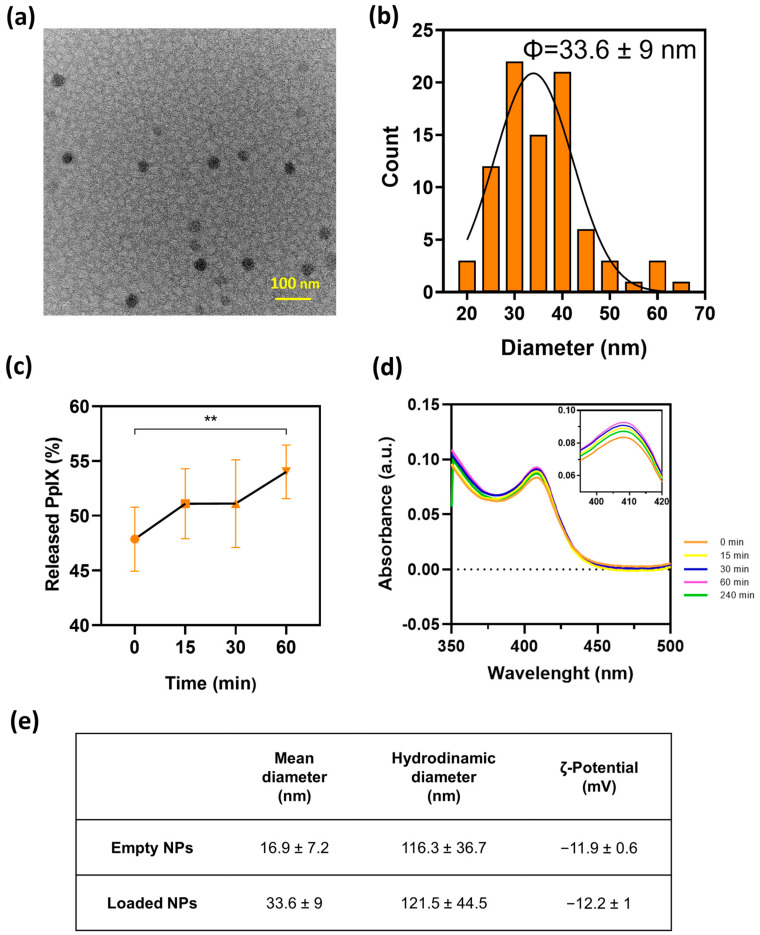
Characterization of PpIX-loaded NPs: (**a**) TEM images of PpIX-loaded NPs. (**b**) Diameter distribution histogram of PpIX-loaded NPs. (**c**) PpIX release kinetics from the particles over a 1 h period in a PBS solution containing Tween^®^20 (2%). Significant difference was found between marked (**) groups (*p* ≤ 0.05). (**d**) Ultraviolet–visible spectrum of PpIX-loaded NPs up to 4 h period-of-release kinetics assay at 37 °C (inset: characteristic absorbance peak of PpIX). (**e**) Measurement of the diameters and ζ potential (pH = 7) of both empty and loaded NPs. Experiments were performed in triplicate (n = 3).

Figure 2a shows the spherical morphology of the resulting PpIX-loaded NPs with mean diameters of 33.6 ± 9 nm (Figure 2b), which are larger than the empty NPs (16.9 ± 7.2 nm), probably attributed to an increased viscosity during photosensitizer entrapment. On the other hand, PpIX release kinetics (Figure 2c) in PBS containing 2 wt.% Tween^®^ 80 described an initial burst release of 47 wt.%, which increased to 54 wt.% in 1 h. This is a positive outcome considering that the photodynamic therapy proposed here is intended to be applied almost instantaneously after applying the released photosensitizer, allowing it to diffuse for 1 h through the bacterial population to favor intra- and interbacterial accumulation. PpIX is protected within the NPs from photobleaching, and upon contact with, for instance, an infected topical wound, it would be released and be prone to subsequent light activation. PpIX protection within the NPs was demonstrated by UV–Vis spectrophotometry (Figure 2d) where the PpIX Soret band at 407 nm was almost unchanged during the time span analyzed (4 h). The slight observed increase over time could be attributed to the contribution of both released PpIX and the remaining PpIX within the NPs as a result of the release. 

Figure 2e shows the hydrodynamic size of the nanoparticles in colloidal suspension. Those measured sizes were larger than those retrieved from the TEM measurements, which is indicative of their tendency for agglomeration. Zeta potential measurements at a neutral pH corroborated those findings because both empty and PpIX-loaded NPs showed electrokinetic potentials of −11.9 ± 0.6 and −12.2 ± 1 mV, respectively, similar to those previously reported for PLGA-based systems [37,38]. Absolute zeta potentials larger than |±30 mV| are an indication of stable colloidal suspensions. However, it is important to point out that the agglomeration was reversible and the NPs were easily suspended after gentle vortexing. The encapsulation efficiency and PpIX loading were 13.7 ± 1.7 wt.% and 0.14 ± 0.09 wt.%, respectively. These results, albeit low, are consistent with those previously found in the literature, with loadings lower than 5% in both PLGA NPs [27,39] and polymeric micelles [16].

**Figure 3:**
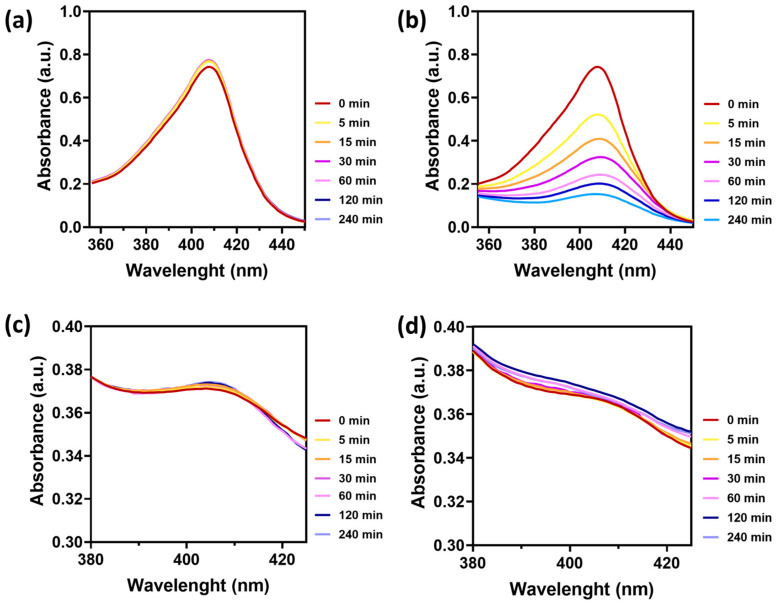
Absorbance spectra of a 10 ppm PpIX solution in DMSO, without (**a**) and with irradiation (**b**) at 0.5 W/cm^2^ for five minutes, irradiating at 0, 5, 15, 30, 60, 120, and 140 min. Absorbance spectra of PpIX-loaded NPs without (**c**) and with irradiation (**d**) under the same conditions. Experiments were performed in triplicate (n = 3).

The photoprotection offered by the polymeric NPs to PpIX was also analyzed by UV–VIS spectrophotometry upon light irradiation. Free PpIX showed a characteristic absorption peak at 407 nm that was stable in the dark for 4 h (Figure 3a), but the signal dramatically decreased upon light irradiation at 0.5 W/cm^2^ (Figure 3b). Successive cycles of light irradiation were used to accelerate photobleaching. In that regard, the absorbance of the free PpIX and the PpIX-loaded nanoparticles was initially measured, and then, both samples were irradiated for 5 min and their absorbance re-measured. Again, this process was repeated by irradiating at 15, 30, 60, 120, and 240 min, and their absorbance was measured (Figure 3b,d). When encapsulated within the polymer NPs, the signal remained constant in the absence (Figure 3c) or presence (Figure 3d) of successive cycles of VIS light with an irradiance of 0.5 W/cm^2^; therefore, the protection performed by the polymeric NPs was demonstrated. It can be seen that the shoulder observed at 407 nm and short periods of incubation (e.g., burgundy curve, time 0) slightly vanish with longer periods of time (e.g., purple curve at 240 min); this effect can be attributed to the loss of the signal due to the photobleached released PpIX; however, the remaining PpIX loaded within the NPs still shows absorbance, which is an indication of its protective effect. It is important to point out that the particles produce scattering and the measured extinction signal represents the contribution of both absorption and scattering. The long-term stability of photosensitizer-loaded PLGA nanoparticles has been previously demonstrated by other authors, which highlights the benefits of using PLGA as carrier [28,40].

### 3.3. Biological Studies

**Figure 4:**
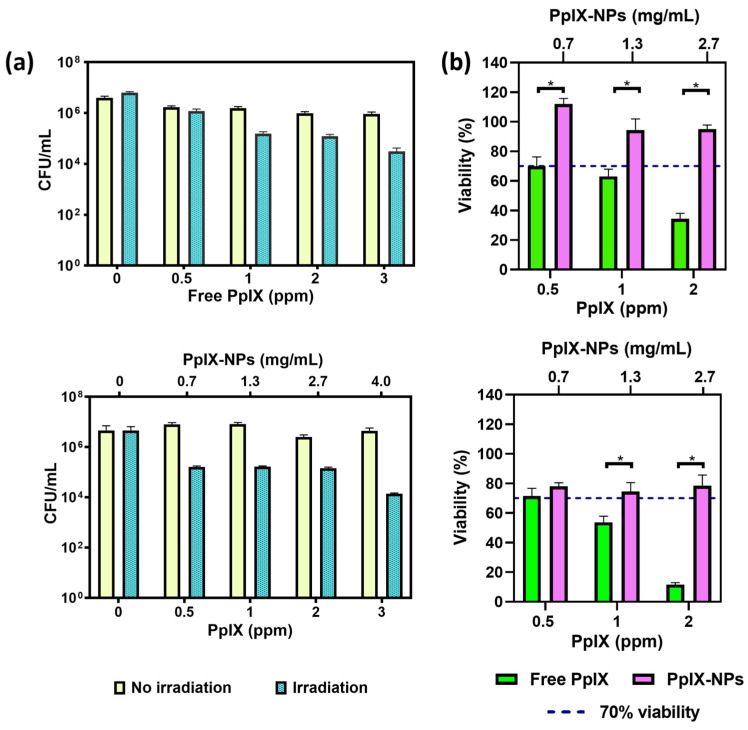
(**a**) Top panel, antibacterial assays against *S. aureus* using free PpIX, and bottom panel, using PpIX-loaded NPs. The quantification of released PpIX is determined by using their drug loading and the release profile from the nanoparticles during the incubation period. (**b**) Top panel, viability assessments of free PpIX and PpIX released from PpIX-loaded NPs on fibroblasts after 1 h incubation, and bottom panel, after 24 h incubation. The values on the upper *x*-axis refer to the columns represented in purple, i.e., the concentration of the samples containing PpIX-loaded NPs. Significant differences were found between marked (*) groups (*p* ≤ 0.001). Empty NP samples exhibited viability exceeding 70%. Experiments were performed in triplicate (n = 3). Viability was calculated by data interpolation assigning 100% viability to control samples (cells without any treatment).

The antimicrobial action of free and encapsulated PpIX against *S. aureus* growth is shown in Figure 4a, where a dose-dependent antimicrobial photodynamic action is depicted for the concentration range assayed (0–3 ppm) for both approaches (free PpIX (top panel) and PpIX-loaded NPs (bottom panel)). In the absence of light, no statistically significant differences with the untreated controls were observed for both approaches, though the irradiation involved a significant decrease in bacteria viability. Specifically, the reduction of two logs in *S. aureus* growth was attained at the highest concentration of PpIX tested (both free and PpIX-loaded NPs). In fact, a slightly higher bactericidal effect (0.5 log) was observed when PpIX-loaded NPs were added to the bacterial cultures (Figure 4a, bottom panel). Therefore, the antimicrobial action was preserved after nanoencapsulation, which is a positive outcome for the biomedical application of the fabricated NPs. In fact, after 1 h, less than 50% of the PpIX was released from the NPs; thus, the superior antimicrobial action of the encapsulated PpIX can be attributed to the protection against degradation in the presence of bacteria. Therefore, equivalent doses of the released photosensitizer were able to reduce the bacterial burden with the additional advantages of protecting it from photobleaching and enhancing its antimicrobial action. The antimicrobial effect of PpIX has been attributed to its ability to generate singlet oxygen upon light irradiation, driven by energy transfer reactions after being excited to a long-lived triplet state [41,42].

For a potential topical application, the cytotoxicities of the free and the encapsulated PpIX were also evaluated (Figure 4b) on fibroblasts. Free PpIX showed a significant dose- and time-dependent cytotoxicity on the tested cell line. When encapsulated, the PpIX showed no cytotoxicity (according to the ISO 10993-5 standard, viability higher than 70%) [43], which can be attributed to a reduced uptake by the eukaryotic cells compared to the free form of the photosensitizer. A reduced size for the free PpIX compared to the PpIX-loaded NPs could be responsible for a superior cellular uptake compared to that for the nanoparticulated carriers.

When a wound occurs, the epidermis becomes disrupted, exposing the dermis and the subcutaneous tissue, and generating a risk of microbial contamination and colonization in these underlying tissues. In the dermis, one of the main types of cells present is fibroblasts [44], which are responsible for repairing damaged tissue [45]. Therefore, it was interesting to understand how a proposed bactericidal treatment could affect this cell line. Other authors have also showed cytotoxic effects of PpIX on fibroblasts, showing a reduction in cell viability of 20–40% at similar concentrations [36,46]. Even higher cytotoxicity on fibroblasts than on melanoma cells has been also reported, exerting a decrease in viability of 45% at higher concentrations (500 ppm) [47]. This previous study also showed larger toxicity for the free photosensitizer than when loaded within polymerosomes, which was attributed to the protective character of the nanoparticles against the intracellular degradation of the photosensitizer. On the other hand, it has been reported that physiological concentrations of PpIX are rapidly converted to heme, though higher concentrations may produce photosensitivity and cytotoxic effects, which could be responsible for the viability decrease observed [14]. Several cytotoxic mechanisms have been reported for PpIX including depolarization of the membrane potential and apoptosis-inducing factor translocation from the mitochondria to the cell nucleus with the subsequent DNA damage [48].

In the potential clinical setting of an infected non-healing wound, after light irradiation, PpIX would generate singlet oxygen in the treatment area, which would be consumed by pathogenic bacteria, and also by some of the cells present in the wound bed (i.e., keratinocytes, fibroblasts, macrophages, etc.). However, the latter would easily regenerate, but thanks to aPDT, this would take place under physiological conditions in a bacteria-free environment due to the removal of the prokaryotic cells.

## 4. Conclusions

Single-emulsion-solvent evaporation allows the successful encapsulation of protoporphyrin IX within PLGA nanoparticles. The protective character, extended release, and increased aqueous solubility of the loaded cargo highlights the benefits of polymer nanoencapsulation of photobleachable photosensitizers. Protoporphyrin-IX-loaded PLGA nanoparticles showed the absence of cytotoxicity on fibroblasts at the highest concentrations tested. They showed high aqueous solubility, photostability, and preserved antimicrobial action upon light irradiation compared to equivalent doses of the free photosensitizer. An initial PpIX burst release from the polymeric nanoparticles might be beneficial to allow a rapid diffusion in the bacterial environment and allow an efficient pathogen photoinactivation, whereas its sustained release could contribute to preventing further recolonization. The proposed PpIX-loaded NPs can overcome the current limitations of light-sensitive hydrophobic photosensitizers also having reduced cytotoxicity against eukaryotic cells, which support their feasibility to be used in biomedical applications.

## Data Availability

Data are contained within the article.
